# Phenotypic diversity in an international Cure VCP Disease registry

**DOI:** 10.1186/s13023-020-01551-0

**Published:** 2020-09-29

**Authors:** Chiseko Ikenaga, Andrew R. Findlay, Michelle Seiffert, Allison Peck, Nathan Peck, Nicholas E. Johnson, Jeffrey M. Statland, Conrad C. Weihl

**Affiliations:** 1 Department of Neurology, Washington University School of Medicine, 660 S. Euclid Avenue, Box 8111, Saint Louis, MO 63110 USA; 2 Cure VCP Disease, Inc., Americus, GA USA; 3 Department of Neurology, Virginia Commonwealth University, Richmond, VA USA; 4 Department of Neurology, University of Kansas, Medical Center, Kansas City, KS USA

**Keywords:** Cure VCP disease patient registry, Multisystem proteinopathy, Valosin-containing protein

## Abstract

**Background:**

Dominant mutations in valosin-containing protein (VCP) gene cause an adult onset inclusion body myopathy, Paget’s disease of bone, and frontotemporal dementia also termed multisystem proteinopathy (MSP). The genotype-phenotype relationships in VCP-related MSP are still being defined; in order to understand this better, we investigated the phenotypic diversity and patterns of weakness in the Cure VCP Disease Patient Registry.

**Methods:**

Cure VCP Disease, Inc. was founded in 2018 for the purpose of connecting patients with VCP gene mutations and researchers to help advance treatments and cures. Cure VCP Disease Patient Registry is maintained by Coordination of Rare Diseases at Sanford. The results of two questionnaires with a 5-point Likert scale questions regarding to patients’ disease onset, symptoms, and daily life were obtained from 59 participants (28 males and 31 females) between June 2018 and May 2020. Independent of the registry, 22 patients were examined at the Cure VCP Disease annual patient conference in 2019.

**Results:**

In the questionnaires of the registry, fifty-three patients (90%) reported that they were with inclusion body myopathy, 17 patients (29%) with Paget’s disease of bone, eight patients (14%) with dementia, two patients (3%) with amyotrophic lateral sclerosis, and a patient with parkinsonism. Thirteen patients (22%) reported dysphagia and 25 patients (42%) reported dyspnea on exertion. A self-reported functional rating scale for motor function identified challenges with sit to stand (72%), walking (67%), and climbing stairs (85%). Thirty-five (59%) patients in the registry answered that their quality of life is more than good. As for the weakness pattern of the 22 patients who were evaluated at the Cure VCP Disease annual conference, 50% of patients had facial weakness, 55% had scapular winging, 68% had upper proximal weakness, 41% had upper distal weakness, 77% had lower proximal, and 64% had lower distal weakness.

**Conclusions:**

The Cure VCP Disease Patient Registry is useful for deepening the understanding of patient daily life, which would be a basis to develop appropriate clinical outcome measures. The registry data is consistent with previous studies evaluating VCP patients in the clinical setting. Patient advocacy groups are essential in developing and maintaining disease registries.

## Background

Multisystem proteinopathy (MSP) is a rare inherited disorder, which affect various organs including the nervous system, skeletal and cardiac muscle, and bone [1, 2]. Mutations in valosin containing protein (*VCP*), *HNRNPA1*, *HNRNPA2B1,* and *SQSTM1* genes have been identified as cause of the disease [3–5]. Among these genes, the *VCP* gene was the first to be detected and its disease-causing mutations account for the majority of patients with this syndrome [6]. Clinically, phenotypes of the VCP-related MSP are heterogeneous even within families, including inclusion body myopathy, Paget’s disease of bone, and frontotemporal dementia [1, 2]. Other phenotypes have been described as amyotrophic lateral sclerosis (ALS), Parkinson’s disease, hereditary spastic paraplegia, Charcot-Marie-Tooth disease, Facial-onset sensory and motor neuronopathy, neuropathy, cardiomyopathy, and cataracts [2, 7–17]. However, few studies inform the phenotypic presentation of VCP-related MSP (VCP disease).

The estimated prevalence of the disease has been reported as 0.66/100,000 population and yet information as to the natural history and phenotypic variability with this expanding phenotype is limited because of the rarity of the disease [18]. Specifically, there have been no reports, which describe the patient experience of living with VCP-related MSP. The Cure VCP Disease Patient Registry was founded to accelerate the development of the cure for this rare disease related to mutations of the *VCP* gene. Since its foundation, the information including patient activity of daily life and quality of life (QOL) has been obtained, which help understanding the varied phenotypes of this disease.

In this article, we combined a retrospective registry cohort, with a cross sectional observational study at the Cure VCP Disease annual patient conference to identify the burden of the disease and better define the phenotypic variability associated with VCP-related MSP.

## Materials and methods

### Organization and study design

Cure VCP Disease, Inc. is a patient advocacy organization formed to drive efforts to find a cure for VCP-related MSP, founded in February 2018. The activities are entirely volunteer led, including outreach to patients, networking with researchers and drug companies, expanding a patient registry, and building awareness in the medical community. In June 2018, the Cure VCP Disease Patient Registry was created to assist in identifying and collecting information about patients. The registry is hosted through Coordination of Rare Diseases at Sanford (CoRDS), a centralized international patient registry for rare diseases. In April 2019, the first Cure VCP Disease patient and caregiver conference was conducted at Washington University in St. Louis.

In this study, we performed a cross-sectional observational study using questionnaires and the results of patient assessments at the first Cure VCP Disease patient conference.

## Participants and data collection

There were 59 participants, who were registered for the Cure VCP Disease Patient Registry between June 2018 and May 2020. Among them, 54 patients were symptomatic and the other five participants were asymptomatic carriers.

At the time of first registration, patients or their legally authorized representative answered to a web-based 81 item questionnaire, which was designed to ask participants’ demographic information and symptoms (Additional file 1: Fig.S1). The information is updated annually. They also took a survey of 69 questions regarding to their mutation type, their diagnoses, how they evaluate their QOL, and cognitive/bulbar/respiratory/truncal/upper extremity/lower extremity functions (Additional file 2: Fig.S2). QOL was rated on a 5-point Likert scale from “poor” to “excellent.” A likert scale was also used to rate function from “no difficulty” to “unable to do”.

Under an independent protocol from the CoRDS registry, 22 symptomatic patients attended the first Cure VCP Disease patient conference in 2019. In the conference, we examined them as to their Medical Research Council (MRC) scale, quantitative manual muscle testing using dynamometry, timed up and go test, Inclusion Body Myositis Functional Rating Scale (IBMFRS), ALS Cognitive Behavioral Screen (ALS-CBS), and Forced vital capacity (FVC) [19–21].

## Statistical analyses

We used Fisher’s exact test to compare categorical variables. For calculating Spearman’s rank correlation coefficient, we used R (version 3.6.1). We considered *p* values less than 0.05 as statistically significant.

## Results

Among the 59 participants registered, 57 participants enrolled by themselves, while two participants were enrolled by their legally authorized representatives for cognitive reasons. Six participants preferred to update their annual information by postal mail, whereas other participants chose to update their information online. Twenty-two patients completed questionnaires, functional tests, and physical exams at an annual patient conference.

### Demographics

**Fig. 1:**
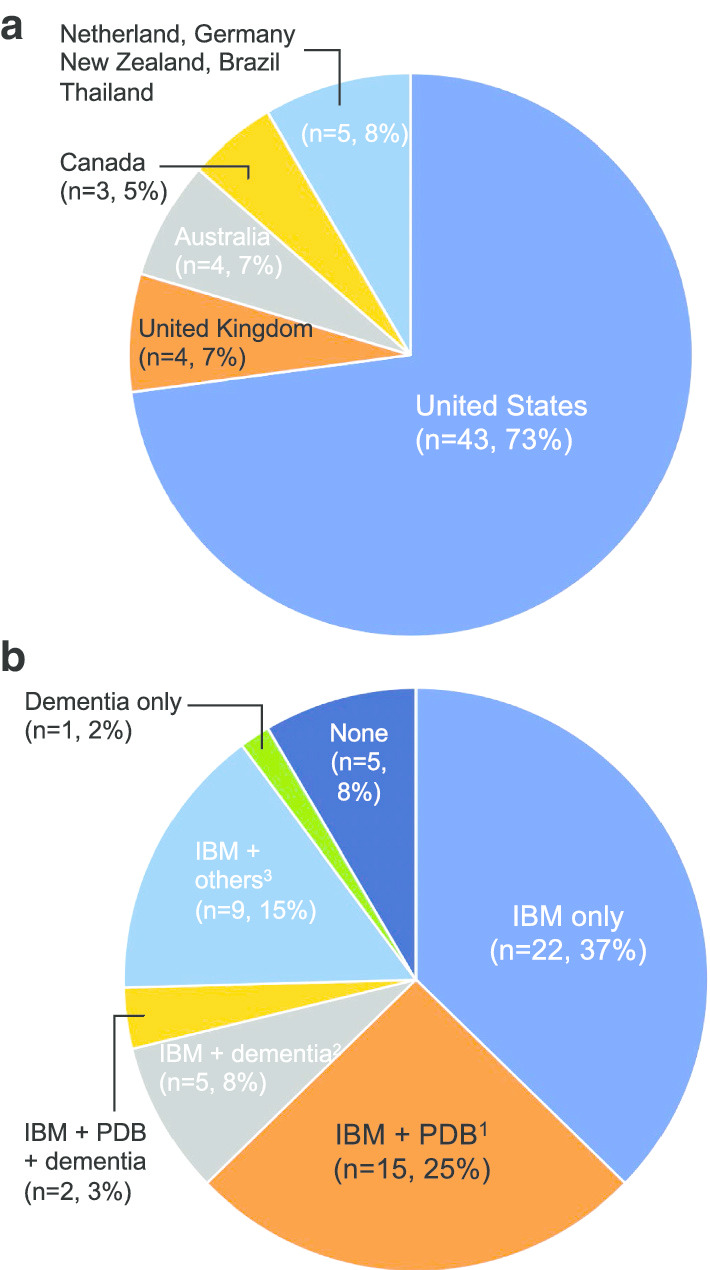
Demographic and phenotypes. Patients from nine countries were registered. Fifty-three patients (90%) were diagnosed with IBM, 17 patients were (29%) with PDB, and eight patients (14%) were with dementia. 1: Two patients were also with peripheral neuropathy. 2: One patient was also diagnosed with cataract and another patient was diagnosed with ALS, cataract, and peripheral neuropathy. 3: Four patients were diagnosed with IBM and cardiomyopathy. One patient was diagnosed with IBM and peripheral neuropathy. Another patient was diagnosed with IBM, peripheral neuropathy, and cataracts. Another patient was diagnosed with IBM and cataracts. Another patient had IBM and Parkinson’s disease. The other patient was diagnosed with IBM, ALS, and peripheral neuropathy. *IBM* inclusion body myopathy, *PDB* Paget’s disease of bone, *ALS* amyotrophic lateral sclerosis

The registry included participants from nine countries (Fig. 1). The registry cohort had slightly more women and 94% were Caucasian (Table 1). Among the 33 patients whose information of mutation types were available, seven mutations (R93C, G125D, R155H, R155C, R159C, R159H, and R191Q) were included.

**Table 1:** Demographics of patients who answered to the questionnaire

	Total participants (n = 59)
Participant sex (M:F)	28:31
Age at onset (n = 41)^a,b,c^	42.4 ± 10.3
Age at onset of muscle weakness (n = 53)^a,b^	40.4 ± 10.0
Age at onset of Paget's disease (n = 13)^a,b^	48.2 ± 10.9
Age at onset of dementia (n = 7)^a,b^	56.7 ± 12.5
Age at diagnosis (n = 55)^a,b^	46.6 ± 10.5
Age at submission^b^	51.7 ± 11.1
Racial categories (n = 55)^a^	
Caucasian	52 (94%)
Asian	2 (4%)
Black or African American	1 (2%)
Ethnicity (n = 44)^a^	
Hispanic or Latino	4 (9%)
Others	40 (91%)
Mutations in VCP gene (n = 33)^a^	
464G>A (R155H)	14 (42%)
463C>T (R155C)	6 (18%)
475C>T (R159C)	4 (12%)
277C>T (R93C)	3 (9%)
476G>A (R159H)	3 (9%)
572G>A (R191Q)	2 (6%)
374G>A (G125D)	1 (3%)

Values are no. (%) unless otherwise indicated

^a^The number of patients in the parenthesis corresponded with that of patients who answered to the questions

^b^Mean ± SD, years

^c^Age at onset refers to the age at onset of the first symptom

### Diagnoses

The reported phenotypes included 53 patients (90%) diagnosed with inclusion body myopathy, 17 patients (29%) with Paget’s disease of bone, eight patients (14%) with dementia, six patients (10%) with peripheral neuropathy, four patients (7%, two patients were with R155H or R191Q mutation in *VCP* gene) with cardiomyopathy, four patients (7%) with cataracts, two patients (3%) with ALS, and one patient (2%) with Parkinson’s disease (Fig. 1). There were five participants without any symptoms, however they had family history of VCP-related MSP and confirmed their own *VCP* gene mutations.

The age at first symptom was 42.4 ± 10.3 and there were 6.5 ± 7.5 from onset to diagnosis (mean ± SD, years). There were 4.9 ± 5.0 (mean ± SD, years) from diagnosis to submission of the questionnaire. The age at onset was around forty on muscle weakness (39.0 ± 9.0 (mean ± SD, years) in patients with R159C) and patients tended to be present with Paget’s disease of bone in mid forty and dementia in mid-fifty.

### Cognitive function

There were 25 patients (42%), who had to read something several times to understand it, 14 patients (24%) had difficulties in planning for and keeping appointments that are not part of their weekly routine, and 16 patients (27%) felt difficulties in learning new tasks or instructions more than once per a week. In the annual Cure VCP Disease conference in 2019, the average score of ALS-CBS was 17.1 out of 20.

### Bulbar, respiratory, and motor functions

**Fig. 2:**
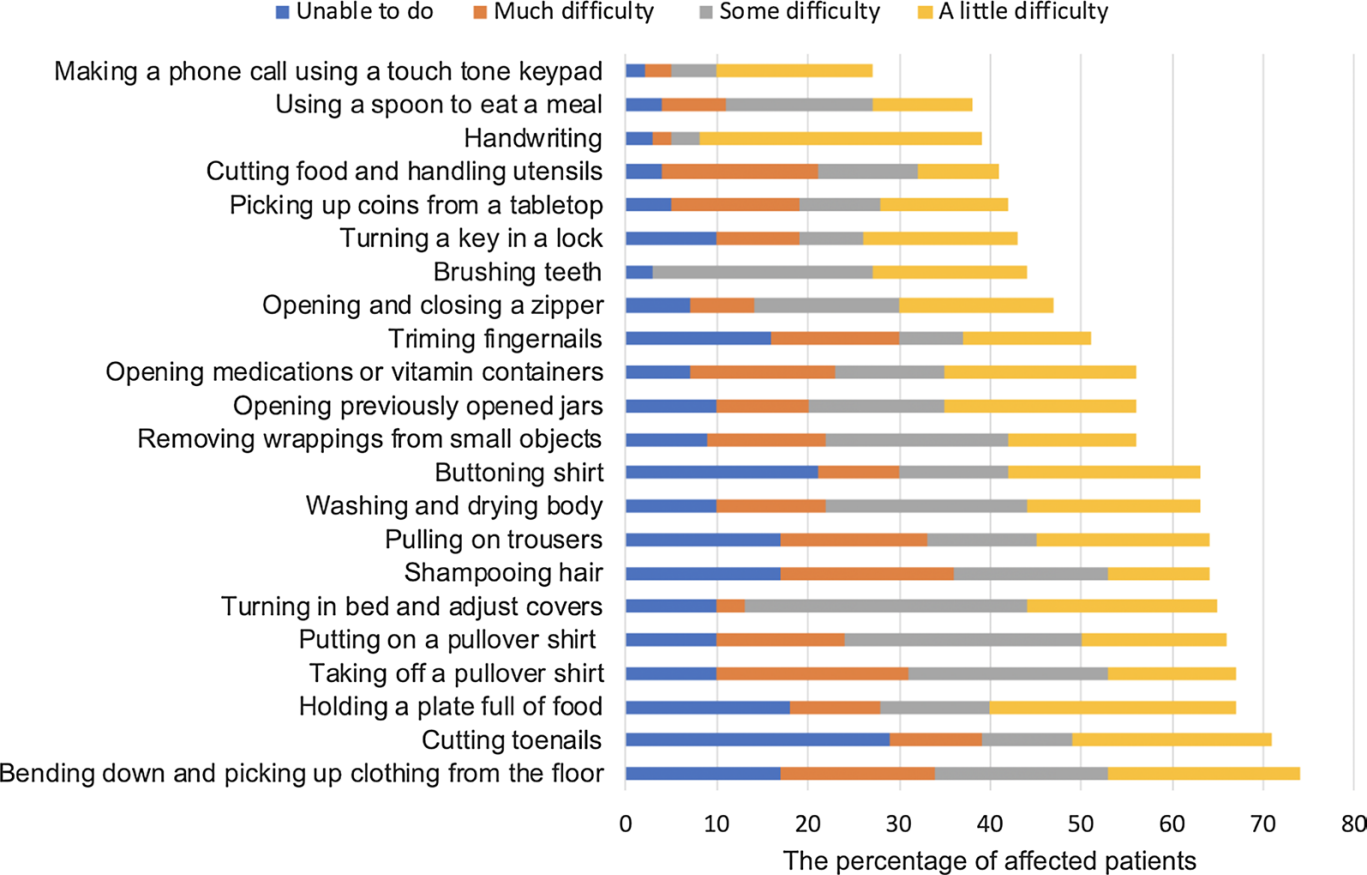
Patients who felt difficulties in the activities of daily life involving upper extremities and trunk. We sent the questionnaire to 59 participants and we were able to get replies from 53 to 58 patients on each question. The figure shows the percentage of patients who felt difficulties in the activities of daily life involving upper extremities and trunk

**Fig. 3:**
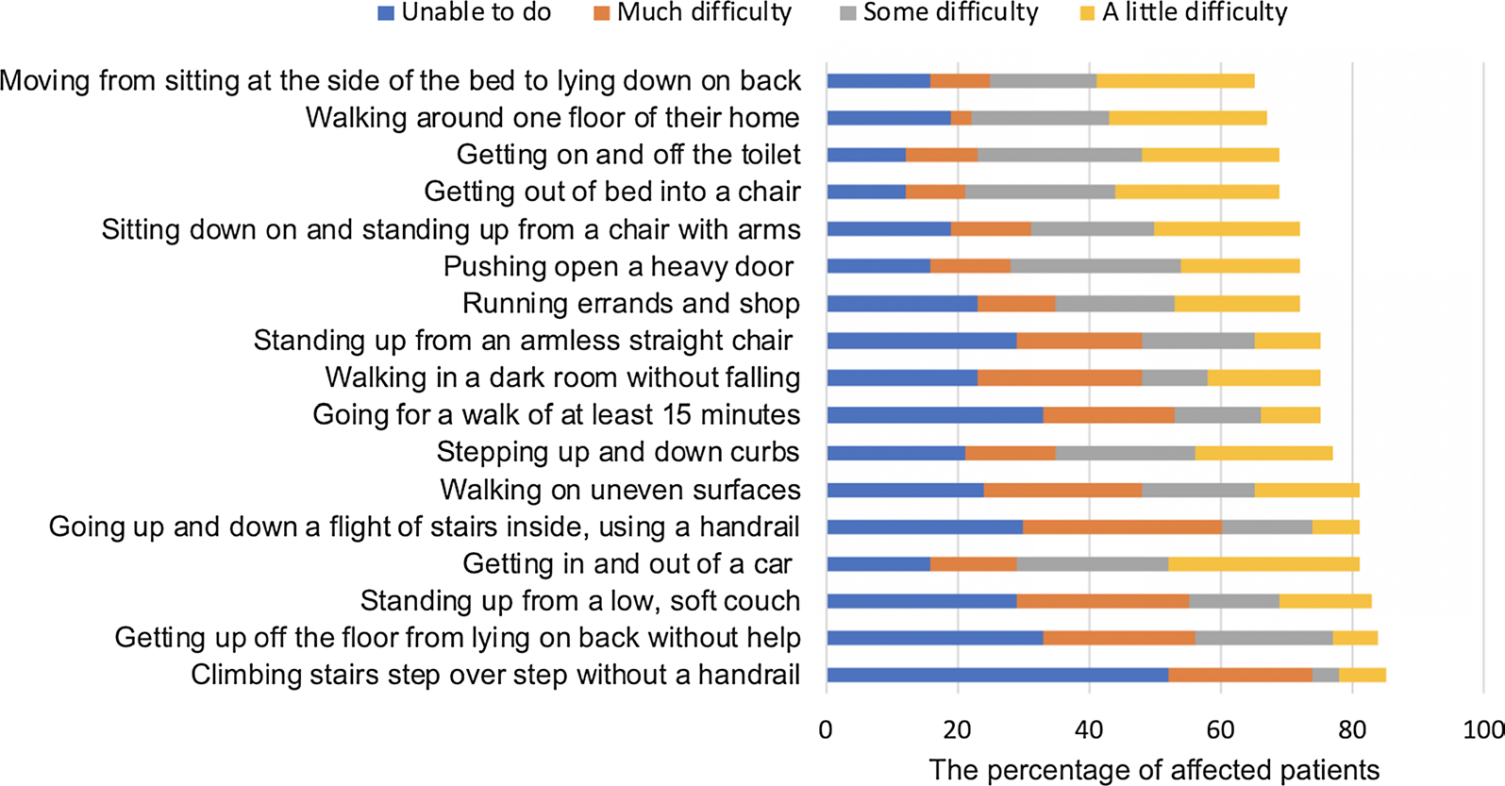
Patients who felt difficulties in the activities of daily life involving lower extremities. We sent the questionnaire to 59 participants and we were able to get replies from 53 to 58 patients on each question. The figure shows the percentage of patients who felt difficulties in the activities of daily life involving lower extremities

Thirteen patients (22%) had experienced occasional choking and one patient needed a modified diet. Seven patients (12%) were also aware of their excessive saliva. Six patients (10%) had detectable speech disturbances and could be intelligible with repeating. Twenty-five patients (42%) noticed their shortness of breath when they were walking, eating, bathing, or dressing. Twelve patients (21%) had orthopnea, seven patients (12%) used some machine which support ventilation or supplementary oxygen, and two patients (3%) were not able to sleep enough despite using extra pillows and ventilatory support machines. Truncal, upper extremity, and lower extremity functions were also assessed by asking their activities in daily life (Fig. 2, Fig. 3).

### Weakness distributions examined at the Cure VCP Disease annual conference in 2019

**Fig. 4:**
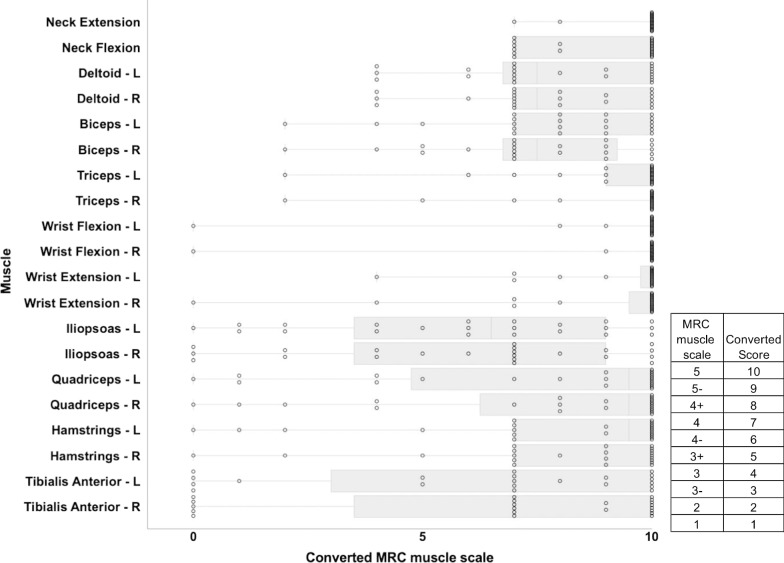
Results of MRC muscle scale. The distribution of weakness on 22 patients was evaluated at the Cure VCP Disease annual conference in 2019 using the MRC scale. MRC muscle scale was converted as indicated. The dots in the figure stand for each patient’s converted score. The line represents the mean value and the boundaries of the box represent the 5–95 percentiles. The bars represent the range. *MRC* Medical Research Council

Among the total 22 patients, who attended the Cure VCP Disease annual conference in 2019, there were 11 patients (50%) with facial weakness and 12 patients (55%) with scapular winging (Table 2). Patients had both upper and lower proximal/distal limbs weakness with a predominance of lower limbs weakness (Fig. 4). There were fifteen patients (68%) with upper proximal weakness, nine patients (41%) with upper distal weakness, 17 patients (77%) with lower proximal weakness, and 14 patients (64%) with lower distal weakness. The distribution of weakness was asymmetric in 12 (55%) patients. Fourteen patients (64%) use walking aids including cane, walker, and wheelchair (Table 2). As for the IBMFRS, the score was 29.0 ± 7.0 (mean ± SD) out of 40 and the score was below 2.5 in the following question: sit to stand, walking, and climbing stairs, which refer to the lower extremity weakness. The forced vital capacity was in the range of 48%–70% of the predicted value in nine patients (41%) (Table 2).

**Table 2:** The result of examination on patients in the conference

	Total participants (n = 22)
Participant sex (M:F)	10:12
Age at onset (n = 19)^a^	43.4 ± 8.9
Age at diagnosis (n = 17)^a^	49 ± 7.9
Age at participation^a^	55.2 ± 9.9
Forced vital capacity, % predicted^b^ (range)	82 ± 25 (48–146)
ALS-CBS^b,c^ (range)	17.1 ± 1.6 (14–20)
Facial weakness	11 (50%)
Weakness of eye closure	7 (32%)
Weakness of lip closure	9 (41%)
Scapular winging	12 (55%)
Bilateral	5 (23%)
Unilateral	7 (32%)
IBM-FRS^b,d^ (range)	29.0 ± 7.0 (9–39)
Timed up and go test^b,e^ (n = 17, s, range)	12.2 ± 6.0 (5.0–27.6)
Walking aid	14 (64%)
Cane	9 (41%)^f^
Walker	11(50%)^g^
Wheelchair	5 (23%)^h^

^a,b^Mean ± SD, years

^c^Amyotrophic Lateral Sclerosis Cognitive Behavioral Screen (ALS-CBS) is a screening tool for frontal lobe dysfunction. We used the cognitive section of ALS-CBS whose total score is 20

^d^Inclusion Body Myositis Functional Rating Scale (IBM-FRS) consists of 10 questions about a patient’s ability to perform daily activities, such as walking, dressing, and handling utensils. Each question was answered using a number 0–4

^e^The Timed Up and Go test is a test for quantifying functional mobility and assessing the fall risk, in which the ability to rise from a seated chair position, walk 3 m, turn, walk back, and sit down is timed

^f^Among the nine patients, five patients always used a cane, while four patients only sometimes used it

^g^Among the eleven patients, two patients always used a walker, while nine patients only sometimes used it

^h^Among the five patients, one patient always used a wheelchair, while four patients only sometimes used it

### Quality of life

Thirty-five patients (59%) answered that their QOL was either excellent (n = 4, 7%), very good (n = 15, 25%), or good (n = 16, 27%). As for everyday physical activities and usual social activities, 31 patients (53%) and 32 patients (54%), respectively, answered that they can carry out these activities completely/mostly/moderately. Mentally, 48 patients (81%) answered that they are in excellent/very good/good condition, although 36 patients (63%) had been sometimes/often/always bothered by emotional problems.

Compared with patients who have isolated muscle weakness, patients with muscle weakness and dementia tended to answer that their QOL is fair or poor (7/26 vs. 6/8, *p* = 0.03), while patients with muscle weakness and Paget’s disease of bone did not refer to significant difference as to QOL (7/26 vs. 9/17, *p* = 0.11). As for the correlation between QOL and other functions, the Spearman’s rank correlation coefficient between QOL and the answers of the following questions were 0.6; push open a heavy door, bend down and pick up clothing from the floor. However, there was no other question which has stronger relevance with QOL than these two questions. As for pain, patients with Paget’s disease of bone rated their own pain as 3.4 ± 1.9 (“0” being no pain and “10” being the worst pain imaginable, mean ± SD), while other symptomatic participants rated their pain as 3.7 ± 2.5 (mean ± SD).

## Discussion

In this study, we describe the physical, mental and social burden on patient daily life in MSP with *VCP* gene mutations using data from a rare disease registry and in person patient conference. These data confirm several features of VCP-related MSP that have been reported by clinical studies. Specifically, as with previous cohorts, there was no significant difference as to the distribution among sexes and onset of symptoms was in the fourth decade [1, 2]. Regarding the mutation type, the two most common mutations were similar to a previous study [22]. The R155H mutation, which is the most common among the patients, has been known to be associated with inclusion body myopathy, Paget’s disease of bone, frontotemporal dementia, and ALS [3, 7]. As for the R159C, which was the third most common in this cohort, later age of onset of myopathy and absence of Paget’s disease of bone was suggested to have relevance [10, 22, 23]. Indeed, there was no patient with Paget’s disease of bone among those with R159C. The age at onset of myopathy was 39.0 ± 9.0 years old in patients with R159C, which was different from the previous study [22].

As for the diagnoses, there was only two patient (3%) who had all the findings of myopathy, Paget’s disease of bone, and frontotemporal dementia. A previous study also showed that patients with the full spectrum of the disease have been reported to make up only 12% of those affected, which suggest that physicians have to make a diagnosis even though some typical clinical feature is lacking [1]. In addition, intrafamilial phenotype variability was also reported, which means that even the patients who have the same variants can present different phenotypes [24]. Compared with a previous clinical study, which described the percent of patients with myopathy 90%, Paget’s disease of bone 42%, and frontotemporal dementia 30%, the percent of patients with dementia in this study was lower [22]. This may be because patients with severe dementia were not able to enroll themselves in this questionnaire-based study. As for the cognitive score of the ALS-CBS, the average score in normal controls was 15.67 ± 2.92 (mean ± SD, 50–59 years) and that in ALS patients was reported to be 14.66 ± 3.54 (mean ± SD, 50–59 years), while other reports that in ALS patients with frontotemporal dementia was 3.7 ± 3.4 in a previous report (mean ± SD) [25, 26]. The average score of the ALS-CBS in the Cure VCP Disease annual meeting was higher than these previous scores, which also suggests that patients with severe dementia were less likely to attend the meeting. Although two patients were able to be enrolled in this study with the help of their legally authorized representatives, it is obvious that caregiver’s support is necessary to follow up this disease, which might cause dementia.

The distribution of muscle weakness varied from limb-girdle pattern to distal dominant or scapuloperoneal pattern. Both questionnaire and examination including IBMFRS/MRC scale suggested involvement of both proximal/distal muscle weakness and predominance of the lower extremity weakness without any muscle sparing. In a previous study, correlation between IBMFRS and MRC scale/fatigue scale/6-min walk test in patients with mutations of the *VCP* gene was detected [27]. The results in this study also support the idea that IBMFRS may correlate with MRC scale in that both suggested the predominance of the lower extremity weakness and that the IBMFRS could be useful for assessing the muscle weakness in the patients with *VCP* gene mutations.

The VCP-related MSP is known to involve not only muscle but also motor neurons and peripheral nerve, which may make the diagnosis difficult to determine [7, 8, 14–16]. With the clinical finding of scapular winging or distal weakness and myopathological finding of rimmed vacuoles as well as TDP-43 immunopositive inclusions, inclusion body myopathy is often confused with GNE myopathy (glucosamine (UDP-*N*-acetyl)-2-epimerase/*N* -acetylmannosamine kinase) or facioscapulohumeral dystrophy [2]. However, compared with GNE myopathy, which also accompany the weakness of tibialis anterior muscles, 45% of the patients accompanied the weakness of quadriceps in this study, which tend to be spared in GNE myopathy [28, 29]. As for the comparison with facioscapulohumeral dystrophy, which accompany the disproportional weakness of biceps and triceps compared to the deltoid and forearm flexors, 64% of the patients showed both deltoid and biceps weakness [30]. In this study, only two patients (9%), who had severe weakness diffusely, showed wrist flexor weakness compared with wrist extensor weakness, which was found around 60% of patients with inclusion body myositis [31]. Although genetic testing is needed for the definite diagnosis of VCP-related MSP, GNE myopathy, and facioscapulohumeral dystrophy, these differences of weakness distribution may help focusing on testing specific gene of each disease.

In French and Spanish cohort, 29% of the patients showed a forced vital capacity under 70% of the predicted value [2]. In a second study, the forced vital capacity as a percentage of the individual’s reference value, was 84.44 ± 29.53 (mean ± SD, %) [27]. The results in this study also suggest the effect on inspiratory muscles and respiratory function should be monitored regularly. Two patients reported cardiomyopathy who had R155H and R191Q mutations in *VCP* gene. Previous reports have identified cardiomyopathy in a patient with an R155C mutation however the correlation between R155H/R191Q and cardiomyopathy is first to be reported [17].

In this study, 60% of patients answered a positive QOL while more than 80% of patients were reporting difficulties in completing specific tasks including climbing stairs without handrail and getting up off the floor from lying on their back without help. The gap is partly because the task can be performed if they use handrail or they are helped by caregivers. In addition, these results seem to suggest that physical inconvenience is not necessarily linked directly to their QOL. All the participants were supported by families or friends, which seem to contribute the positive QOL at least partly.

This study has several limitations. First, the nature of VCP-related MSP is that as a patient progresses, new phenotypes can emerge. For example, dementia is a later onset feature in most patients. Therefore, the reliability of this cross-sectional study for disease phenotypes throughout a patient’s lifetime is limited. Follow up of the patients is needed to clarify the natural history of this disease. Second, the number of patients who enrolled in the registry is limited. Researchers and patients need to increase awareness of advocacy groups and their activities. This is a significant challenge for rare diseases in which the recruitment of single patient can influence prevalence. The Cure VCP Disease, Inc. registry is limited as it is written in English only and biases enrollment. Future goals will be to prepare a homepage and questionnaire written in other languages. Third, the clinical information in the registry is patient or caregiver reported and should be cautiously interpreted. Performing assessments at an annual meeting that represents patients in the registry can help to validate patient reported findings. We are reassured by the fact that our data so closely matches previous cohort studies. Due to the different structure of the patient meeting consents, which was performed under a local institutional review board, and the structure of the registry consents, which was housed and curated by CoRDS, we were unable to link patients in each cohort. Therefore, the participants in each cohort should be treated as independent cohorts that may have an overlap of patients. For future patient conferences, we plan to include language within the consent that will enable us to link these participants from the registry to a patient visit.

## Conclusion

The Cure VCP Disease Patient Registry provides the necessary tools for remote recruitment and enrollment. Sharing information between patients and physician/researchers through the registry will accelerate the establishment of appropriate clinical outcome measurement and/or biomarkers, and implementation of physical therapy and disease modifying therapies in future.

## Supplementary information

**Additional file 1: Figure S1** CoRDS Registry questionnaire. Questionnaire composed of 81 questions regarding to contact information, socio-demographic information, diagnosis, family history, and quality of life.

**Additional file 2: Figure S2** Cure VCP Disease, Inc. questionnaire. Questionnaire composed of 69 questions regarding to mutation type, diagnoses, quality of life, and cognitive/bulbar/respiratory/truncal/upper extremity/lower extremity functions.

## Data Availability

Patient registry data was provided by the Coordination of Rare Diseases at Sanford, a rare disease registry sponsored by Sanford Health. The deidentified patient data obtained at the Cure VCP Disease annual patient conference in 2019 are available from the corresponding author, upon reasonable request.

## Abbreviations

VCP: Valosin-containing protein
MSP: Multisystem proteinopathy
HNRNPA1: Heterogeneous nuclear ribonucleoprotein A1
HNRNPA2B1: Heterogeneous nuclear ribonucleoproteins A2/B1
SQSTM1: Sequestosome 1
QOL: Quality of life
CoRDS: Coordination of Rare Diseases at Sanford
MRC: Medical Research Council
IBMFRS: Inclusion Body Myositis Functional Rating Scale
ALS-CBS: Amyotrophic Lateral Sclerosis Cognitive Behavioral Screen
FVC: Forced vital capacity
SD: Standard deviation
GNE: Glucosamine (UDP-*N*-acetyl)-2-epimerase/*N*-acetylmannosamine kinase
