# Supplementary material for: Phenotypic diversity in an international Cure VCP Disease registry
Source: Orphanet J Rare Dis. 2020 Sep 29;15:267. doi: 10.1186/s13023-020-01551-0 (PMC7523394; doi:10.1186/s13023-020-01551-0)
Supplement: Supplementary file 1 — Additional file 1: Figure S1 CoRDS Registry questionnaire. Questionnaire composed of 81 questions regarding to contact information, socio-demographic information, diagnosis, family history, and quality of life. [file 13023_2020_1551_MOESM1_ESM.pdf]

CoRDS Registry

Coordination of Rare Diseases at Sanford

Instructions

Thank you for taking the time to enroll with the CoRDS Registry. This questionnaire:

- Takes 10 - 20 minutes to complete
- Will refer to the person with the rare or unknown diagnosis as "**the participant**"
- Can be updated at any time by logging in to the CoRDS online portal or by contacting CoRDS personnel

CoRDS personnel will contact you annually to update your questionnaire.

If you have any questions while completing this form, please contact CoRDS at (877) 658 – 9192 during business hours, 8:30am-5:00pm (CST) Monday through Friday. If you need assistance after business hours, please leave a message or email cords@sanfordhealth.org.

*For accurate data curation, please remember to write legibly. Thank you.

1. Today's Date (MM/DD/YYYY):

2. Who is completing this questionnaire?

☐ I am enrolling myself (You must be over the age of 18 to provide information for the registry)

☐ I am enrolling my child (You must be the participant's parent or legal guardian to provide information for the registry)

☐ I am enrolling an adult who is not cognitively able to enroll (You must be the participant's legally authorized representative (LAR) to provide information for the registry)

Permissions & Data Sharing

By participating in CoRDS, your de-identified information will be shared with researchers who access the CoRDS Registry. Below are options that allow you to share your data with other entities. In the following questions, please select how you want your data shared. Please complete this section before moving on.

3. I give permission to CoRDS to contact me about participating in future research studies:

☐ Yes ☐No ☐Don't Know

4. I give permission to CoRDS to contact me about donating a sample of blood, tissue, or other biospecimen for research in the future:

☐ Yes ☐No ☐Don't Know

5. I give permission to CoRDS to provide a subset of de-identified information to other databases collecting information on rare diseases in order to avoid a duplication of efforts and to increase knowledge:

☐ Yes ☐No

Participant Information

**6. First Name:**	**7. Middle Name:**	**8. Last Name:**

☐ Check if the legal given name (as per birth certificate) of the participant is the same as indicated above

Legal given name of the participant (as per birth certificate)

**9. First Name:**	**10. Middle Name:**	**11. Last Name:**
**12. Date of Birth**:	**13. City of Birth:**	**14. Country of Birth:**
**15. Address 1:**		
**16. Address 2:**		
**17. City:**	**18. State**	**Province**

19. Zip/Postal Code	20. Country	
**21. Email Address:**		
**22. Phone Number:**		

Parent / Legally Authorized Representative (LAR) Information

Please complete this section if you are the participant's parent/guardian (**participant must be under the age of 18**) or legally authorized representative (**participant is not cognitively able to enroll).**

**23. First Name:**		
**24. Middle Name:**		
**25. Last Name:**		
**26. Primary Telephone Number:**		
**27. Email Address:**		
☐ Check if the address is the same as the participant's, then skip to next section		
**28. Address 1:**		
**29. Address 2:**		
**30. City:**	**31. State**	**Province**
**32. Zip/Postal Code:**	**33. Country:**	

Secondary Contact

Please provide information for an individual that we may contact in the event that we are unable to reach you.

34. Relationship to Secondary Contact:

☐ Family Member ☐ Spouse/Partner
☐ Friend ☐ Other

**35. If you selected "Other" above, please specify: ________________________**	
**36. First Name:**	
**37. Middle Name:**	
**38. Last Name:**	
**39. Primary Telephone Number:**	
**40. Email Address:**	
☐ Check if the address is the same as the participant's, then skip to next section	
**41. Address 1:**	
**42. Address 2:**	
**43. City:**	
**44. State**	**Province**
**45. Zip/Postal Code:**	**46. Country:**

Enrollment, Contact & Communication Preferences

**47. How would you like to complete your annual updates?**	☐Online	☐Postal mail	
**48. How would you like CoRDS to contact you?**	☐Email	☐Phone	☐Postal mail

49. Special Communication Needs: Do you (the person completing this form) have any special communication needs? Please select all that apply, or describe in the space provided.

☐ No special needs - both spoken and written language are acceptable
☐ Sign language required
☐ Spoken language preferred
☐ Written language preferred
☐ Other

50. If you selected "Other" above, please specify: ____

Participant Socio-demographic Information

Please provide information about the participant's background and diagnosis in the following sections.

**51. Sex:**	☐ Female	☐ Male	☐ Transsexual	☐ Unknown	☐ Other
**52. Sex at Birth:**	☐ Female	☐ Male	☐ Transsexual	☐ Unknown	☐ Other

53. Race:

☐ American Indian or Alaska Native
☐ Asian - Asian Indian
☐ Asian - Chinese
☐ Asian - Filipino
☐ Asian - Japanese
☐ Asian - Korean
☐ Asian - Vietnamese
☐ Asian - Other Asian
☐ Black or African American
☐ Pacific Islander – Native Hawaiian
☐ Pacific Islander - Guamanian
☐ Pacific Islander - Chamorro
☐ Pacific Islander- Samoan
☐ Pacific Islander - Other Pacific Islander
☐ White
☐ Other/Unknown/Refuse to Answer

54. Ethnicity:

☐ Ashkenazi Jewish
☐ French Canadian
☐ Hispanic or Latino - Central American
☐ Hispanic or Latino - Cuban
☐ Hispanic or Latino - Dominican (Republic)
☐ Hispanic or Latino - Mexican
☐ Hispanic or Latino - Puerto Rican
☐ Hispanic or Latino - South American
☐ Hispanic or Latino - Other Latin American
☐ Hispanic or Latino - Other Hispanic/Latino/Spanish
☐ Not Hispanic or Latino
☐ Unknown/No answer
☐ Other

55. If you selected "Other" above, please specify: ___________________________________

56. Is the participant still living? ☐ Yes ☐ No ☐ Don't know

57. If you selected "No" above, please indicate date of death (MM/DD/YYYY): _______________

Diagnosis

58. For genetic rare diseases, is the participant an unaffected carrier of the rare disease?

☐ Yes ☐ No ☐ Unknown

59. If you selected "Yes" above, please list the rare disease for which the participant is a carrier for.

60. Rare Disease Diagnosis: Please list all rare disease diagnoses.

61. Rare Disease Symptoms: Please list symptoms of rare disease diagnoses. Separate with commas.

62. Undiagnosed: If no clinical diagnosis has been made, please list symptoms. Separate with commas.

63. Other Diagnoses: Please list non-rare diagnoses. Separate with commas.

64. Age at Diagnosis: ☐ Prenatal ☐ At birth ☐ Age ☐ Unknown ☐ N/A

65. If you selected "Age" above, please indicate age: (years and/or months) _____

66. Age at First Symptom: ☐ Prenatal ☐ At birth ☐ Age ☐ Unknown ☐ N/A

67. If you selected "Age" above, please indicate age: (years and/or months) _____

68. How was the rare diagnosis determined? Select all that apply.

☐ Genetic Laboratory Analysis
☐ Histology
☐ Imaging – CT
☐ Imaging – MRI
☐ Imaging – PET
☐ Physical Examination
☐ Unknown
☐ Other

69. If you selected "Other" above, please specify:
____

70. Where was the diagnosis made?

Hospital / Institution: ____

City: ____

State or Province: ____

Country: ____

Family History

71. Which family members also have the participant's rare disease? Select all that apply.

☐ None
☐ Mother
☐ Father
☐ Brother
☐ Half-brother
☐ Sister
☐ Half-sister
☐ Daughter
☐ Son
☐ Maternal Grandfather
☐ Paternal Grandfather
☐ Maternal Grandmother
☐ Unknown
☐ Paternal Grandmother
☐ Maternal Aunt
☐ Paternal Aunt
☐ Maternal Uncle
☐ Paternal Uncle
☐ Maternal Cousin
☐ Paternal Cousin
☐ Granddaughter
☐ Grandson
☐ Niece
☐ Nephew

Quality of Life

72. In general, would the participant say his/her health is…

☐ Excellent	☐ Very good	☐ Good	☐Fair	☐ Poor

73. Does the participant's health now limit him/her in doing vigorous activities?

☐ Never	☐ Rarely	☐ Sometimes	☐ Often	☐ Always

74. How much did pain interfere with the participant's enjoyment of life?

☐ Never	☐ Rarely	☐ Sometimes	☐ Often	☐ Always

75. How often does the participant feel tired?

☐ Never	☐ Rarely	☐ Sometimes	☐ Often	☐ Always

76. The participant feels depressed…

☐ Never	☐ Rarely	☐ Sometimes	☐ Often	☐ Always

Clinical Research Participation & Biospecimens

77. Has the participant *previously participated in any clinical trials* related to their rare disease?

☐ Yes ☐ No ☐ Don't know

78. Does the participant *currently participate in any clinical trials* related to their rare disease?

☐ Yes ☐ No ☐ Don't know

79. Has the participant *previously donated a sample* of blood, tissue, or other biospecimen for research?

☐ Yes ☐ No ☐ Don't know

80. If Yes:

Type of biospecimen:

☐ Blood ☐ Tissue
☐ Other bodily fluid ☐ Urine
☐ Saliva/Cheek Swab ☐ Unknown

81. Location of biospecimen donation:

☐ Check here if location unknown

Hospital/ Institution: ________________________________

City: ____

State or Province: __________________________________

Country:____

Thank you for your participation!

Questions?

CoRDS Personnel

Sanford Research

2301 East 60th Street North

Sioux Falls, South Dakota 57104

Phone (toll-free)**:** 1 (877) 658-9192

Email: CoRDS@sanfordhealth.org
