# Supplementary material for: Phenotypic diversity in an international Cure VCP Disease registry
Source: Orphanet J Rare Dis. 2020 Sep 29;15:267. doi: 10.1186/s13023-020-01551-0 (PMC7523394; doi:10.1186/s13023-020-01551-0)
Supplement: Supplementary file 2 — Additional file 2: Figure S2 Cure VCP Disease, Inc. questionnaire. Questionnaire composed of 69 questions regarding to mutation type, diagnoses, quality of life, and cognitive/bulbar/respiratory/truncal/upper extremity/lower extremity functions. [file 13023_2020_1551_MOESM2_ESM.pdf]

Instructions

Thank you for taking the time to enroll with the CoRDS Registry. This module will ask you questions specific to your diagnosis. The questions below were developed in partnership with Cure VCP Disease, Inc. Please note, this module:

- Takes approximately 10-20 minutes to complete
- Will refer to the person with the diagnosis as "**the participant**"
- Can be updated at any time by logging in to the CoRDS online portal or by contacting CoRDS personnel

If you have any questions while completing this form, please contact CoRDS at (877) 658-9192 during business hours, 8:30am-5:00pm (CST) Monday through Friday. If you need assistance after business hours, please leave a message or email cords@sanfordhealth.org.

Permissions & Data Sharing

I give permission to CoRDS to provide my information that may or may not be identifiable to the following Patient Advocacy Group (PAG) for non-research purposes.

☐ Cure VCP Disease, Inc. ☐ I do not give my permission

Diagnosis

1. What was the age of onset for the following symptoms or conditions of the participant's IBMPFD?

**Dementia**	Age (years): ________________	☐ Not applicable
**Muscle weakness**	Age (years): ________________	☐ Not applicable
**Paget's**	Age (years): ________________	☐ Not applicable

2. Does the participant know their gene mutation?

☐ No, I have not been tested ☐ Yes, but I can't remember what it is

☐ No, I have been tested but no mutation was found	☐ Yes
**3. If yes, please indicate which mutation the participant possesses.**	
☐ p.Arg93Cys c.277C>T	☐ Arg159His 476G>A
☐ Ala232Glu 695C>A	☐ Arg191Gln 572G>A
☐ Arg155Cys 463C>T	☐ Arg95Gly 283C>G
☐ Arg155His 464G>A	☐ Asn387His 1159A>C
☐ Arg155Pro 464G>C	☐ Leu198Trp 593T>G
☐ Arg159Cys 475C>T	☐ Other
**Please specify other:**	
**4. Please select any of the following which a physician told the participant that he/she has:**	
☐ Amyotrophic Lateral Sclerosis (ALS)	☐ Paget's Disease
☐ Cardiomyopathy	☐ Parkinson's
☐ Cataracts	☐ Peripheral Neuropathy
☐ Dementia	☐ None
☐ Myopathy	☐ Other
**If "Other", please specify:**	
**5. The participant's Paget's disease current diagnosis is:**	
☐ Bone pain	☐ None/Normal
☐ Elevated blood test results	☐ Visible on X-ray
**6. Which of the following does the participant have a family history of?**	
☐ ALS or Lou Gehrig Disease	☐ Muscle Weakness
☐ Dementia	☐ Paget's Disease of the Bone
☐ Inclusion Body Myopathy	☐ None

**7. If the participant does have a family history, please list their relationship and their known symptoms/diagnosis. (Please indicate if the relationship is Paternal or Maternal)**

Supplemental Oxygen

**8. Does the participant use supplemental oxygen or BiPAP/CPAP for breathing?**	
☐ BiPap/CPAP	☐ Supplemental oxygen
☐ No assistance for breathing	☐ Other
**Please specify other:**	

Global Health

**9. In general, the participant's quality of life is:**				
☐ Excellent	☐ Very good	☐ Good	☐ Fair	☐ Poor
**10. In general, how would the participant rate their mental health, including mood and ability to think?**				
☐ Excellent	☐ Very good	☐ Good	☐ Fair	☐ Poor
**11. In general, how would the participant rate their satisfaction with social activities and relationships?**				
☐ Excellent	☐ Very good	☐ Good	☐ Fair	☐ Poor
**12. In general, please rate how well the participant carries out usual social activities and roles. (This includes activities at home, at work and in their community, and responsibilities as a parent, child, spouse, employee, friend, etc.)**				
☐ Excellent	☐ Very good	☐ Good	☐ Fair	☐ Poor
**13. To what extent is the participant able to carry out everyday physical activities such as walking, climbing stairs, carrying groceries, or moving a chair?**				
☐ Completely	☐ Mostly	☐ Moderately	☐ A little	☐ Not at all

14. How often has the participant been bothered by emotional problems such as feeling anxious, depressed or irritable?				
☐ Never	☐ Rarely	☐ Sometimes	☐ Often	☐ Always

15. How would the participant rate their pain on average? (With "0" being no pain and "10" being the worst pain imaginable)										
☐ 0	☐ 1	☐ 2	☐ 3	☐ 4	☐ 5	☐ 6	☐ 7	☐ 8	☐ 9	☐ 10

Cognition Function				
**16. In the past 7 days, the participant had to read something several times to understand it…**				
☐ Never	☐ Rarely (once)	☐ Sometimes (2-3 times)	☐ Often (once a day)	☐ Very often (several times a day)
**17. In the past 7 days, the participant's thinking was slow…**				
☐ Never	☐ Rarely (once)	☐ Sometimes (2-3 times)	☐ Often (once a day)	☐ Very often (several times a day)
**18. In the past 7 days, the participant had to work really hard to pay attention, otherwise they would make a mistake…**				
☐ Never	☐ Rarely (once)	☐ Sometimes (2-3 times)	☐ Often (once a day)	☐ Very often (several times a day)
**19. In the past 7 days, the participant had trouble concentrating…**				
☐ Never	☐ Rarely (once)	☐ Sometimes (2-3 times)	☐ Often (once a day)	☐ Very often (several times a day)
**20. How much difficulty does the participant currently have reading and following complex instructions (e.g., directions for a new medication)?**				
☐ None	☐ A little	☐ Somewhat	☐ A lot	☐ Cannot do
**21. How much difficulty does the participant currently have planning for and keeping appointments that are not part of your weekly routine (e.g. a therapy or doctor appointment, or a social gathering with friends and family)?**				

☐ None	☐ A little	☐ Somewhat	☐ A lot	☐ Cannot do
**22. How much difficulty does the participant currently have managing their time to do most of their daily activities?**				
☐ None	☐ A little	☐ Somewhat	☐ A lot	☐ Cannot do
**23. How much difficulty does the participant currently have learning new tasks or instructions?**				
☐ None	☐ A little	☐ Somewhat	☐ A lot	☐ Cannot do

Functions				
**Please rate the following categories according to the participants abilities:**				
**24. Swallowing**				
☐ Normal	☐ Early eating problems or occasional choking	☐ Dietary consistency changes (e.g., need more soups)	☐ Frequent choking	☐ Needs tube feeding
**25. Turn in Bed and Adjust Covers**				
☐ Normal	☐ Somewhat slow and clumsy, but no help needed	☐ Can turn alone or adjust sheets, but with great difficulty	☐ Can initiate, but not turn or adjust sheets alone	☐ Unable or requires total assistance
**26. Handwriting (with dominant hand prior to IBMPFD onset) (ALSFRS, IBMFRS)**				
☐ Normal	☐ Slow or sloppy; all words are legible	☐ Not all words are legible	☐ Able to grip pen but unable to write	☐ Unable to grip pen
**27. Cutting food and handling utensils (ALSFRS, IBMFRS)**				
☐ Normal	☐ Somewhat slow and clumsy, but no help needed	☐ Can cut most foods although clumsy and slow; some help needed	☐ Food must be cut by someone, but can still feed slowly	☐ Needs to be fed
**28. Speech**				

☐ Normal Speech Processes	☐ Detectable Speech Disturbances	☐ Intelligible with repeating	☐ Speech combined with nonvocal communication	☐ Loss of useful speech
**29. Salivation**				
☐ Normal	☐ Slight but define excess of saliva in mouth; may have minimal drooling	☐ Moderately excessive saliva; may have minimal drooling	☐ Marked excess of saliva with some drooling	☐ Marked drooling; requires constant tissue or handkerchief
**30. Dyspnea – Shortness of breath**				
☐ None	☐ Occurs when walking	☐ Occurs with 1 or more of the following eating, bathing, dressing (ADL)	☐ Occurs at rest, difficulty breathing when either sitting or lying	☐ Significant difficulty, considering using mechanical respiratory support
**31. Orthopnea – Shortness of breath when lying flat**				
☐ None	☐ Some difficulty sleeping at night due to shortness of breath. Does not use more than 2 pillows	☐ Need extra pillow in order to sleep (more than 2)	☐ Can only sleep sitting up	☐ Unable to sleep
**32. Respiratory Insufficiency**				
☐ None	☐ Intermittent use of BiPAP	☐ Continuous use of BiPAP	☐ Continuous use of BiPAP during the day and night	☐ Invasive mechanical ventilation by intubation or tracheostomy

**Lower Extremity Function**				
**33. How able is the participant to get on and off the toilet?**				
☐ Without any difficulty	☐ With a little difficulty	☐ With some difficulty	☐ With much difficulty	☐ Unable to do

34. How able is the participant to step up and down curbs?				
☐ Without any difficulty	☐ With a little difficulty	☐ With some difficulty	☐ With much difficulty	☐ Unable to do
**35. How able is the participant to get in and out of a car?**				
☐ Without any difficulty	☐ With a little difficulty	☐ With some difficulty	☐ With much difficulty	☐ Unable to do
**36. How able is the participant to get out of bed into a chair?**				
☐ Without any difficulty	☐ With a little difficulty	☐ With some difficulty	☐ With much difficulty	☐ Unable to do
**37. How able is the participant to push open a heavy door?**				
☐ Without any difficulty	☐ With a little difficulty	☐ With some difficulty	☐ With much difficulty	☐ Unable to do
**38. How able is the participant to run errands and shop?**				
☐ Without any difficulty	☐ With a little difficulty	☐ With some difficulty	☐ With much difficulty	☐ Unable to do
**39. How able is the participant to get up off the floor from lying on their back without help?**				
☐ Without any difficulty	☐ With a little difficulty	☐ With some difficulty	☐ With much difficulty	☐ Unable to do
**40. How able is the participant to go for a walk of at least 15 minutes?**				
☐ Without any difficulty	☐ With a little difficulty	☐ With some difficulty	☐ With much difficulty	☐ Unable to do
**41. How much DIFFICULTY does the participant currently have standing up from an armless straight chair (e.g., dining room chair)?**				
☐ No difficulty	☐ A little difficulty	☐ Some difficulty	☐ A lot of difficulty	☐ Can’t do
**42. How much DIFFICULTY does the participant currently have sitting down on and standing up from a chair with arms?**				

☐ No difficulty	☐ A little difficulty	☐ Some difficulty	☐ A lot of difficulty	☐ Can't do
**43. How much DIFFICULTY does the participant currently have moving from sitting at the side of the bed to lying down on their back?**				
☐ No difficulty	☐ A little difficulty	☐ Some difficulty	☐ A lot of difficulty	☐ Can't do
**44. How much DIFFICULTY does the participant currently have standing up from a low, soft couch?**				
☐ No difficulty	☐ A little difficulty	☐ Some difficulty	☐ A lot of difficulty	☐ Can't do
**45. How much DIFFICULTY does the participant currently have going up and down a flight of stairs inside, using a handrail?**				
☐ No difficulty	☐ A little difficulty	☐ Some difficulty	☐ A lot of difficulty	☐ Can't do
**46. How much DIFFICULTY does the participant currently have walking on uneven surfaces (e.g., grass, dirt road or sidewalk)?**				
☐ No difficulty	☐ A little difficulty	☐ Some difficulty	☐ A lot of difficulty	☐ Can't do
**47. How much DIFFICULTY does the participant currently have walking around one floor of their home?**				
☐ No difficulty	☐ A little difficulty	☐ Some difficulty	☐ A lot of difficulty	☐ Can't do
**48. How much DIFFICULTY does the participant currently have climbing stairs step over step without a handrail? (alternating feet)?**				
☐ No difficulty	☐ A little difficulty	☐ Some difficulty	☐ A lot of difficulty	☐ Can't do
**49. How much DIFFICULTY does the participant currently have walking in a dark room without falling?**				

☐ No difficulty	☐ A little difficulty	☐ Some difficulty	☐ A lot of difficulty	☐ Can't do

Upper Extremity Function

50. How able is the participant to turn a key in a lock?

☐ Without any difficulty	☐ With a little difficulty	☐ With some difficulty	☐ With much difficulty	☐ Unable to do

51. How able is the participant to brush their teeth?

☐ Without any difficulty	☐ With a little difficulty	☐ With some difficulty	☐ With much difficulty	☐ Unable to do

52. How able is the participant to make a phone call using a touch tone key-pad?

☐ Without any difficulty	☐ With a little difficulty	☐ With some difficulty	☐ With much difficulty	☐ Unable to do

53. How able is the participant to pick up coins from a table top?

☐ Without any difficulty	☐ With a little difficulty	☐ With some difficulty	☐ With much difficulty	☐ Unable to do

54. How able is the participant to write with a pen or pencil?

☐ Without any difficulty	☐ With a little difficulty	☐ With some difficulty	☐ With much difficulty	☐ Unable to do

55. How able is the participant to open and close a zipper?

☐ Without any difficulty	☐ With a little difficulty	☐ With some difficulty	☐ With much difficulty	☐ Unable to do

56. How able is the participant to wash and dry their body?

☐ Without any difficulty	☐ With a little difficulty	☐ With some difficulty	☐ With much difficulty	☐ Unable to do

57. How able is the participant to shampoo their hair?

☐ Without any difficulty	☐ With a little difficulty	☐ With some difficulty	☐ With much difficulty	☐ Unable to do
**58. How able is the participant to open previously opened jars?**				
☐ Without any difficulty	☐ With a little difficulty	☐ With some difficulty	☐ With much difficulty	☐ Unable to do
**59. How able is the participant to hold a plate full of food?**				
☐ Without any difficulty	☐ With a little difficulty	☐ With some difficulty	☐ With much difficulty	☐ Unable to do
**60. How able is the participant to pull on trousers?**				
☐ Without any difficulty	☐ With a little difficulty	☐ With some difficulty	☐ With much difficulty	☐ Unable to do
**61. How able is the participant to button their shirt?**				
☐ Without any difficulty	☐ With a little difficulty	☐ With some difficulty	☐ With much difficulty	☐ Unable to do
**62. How able is the participant to trim their fingernails?**				
☐ Without any difficulty	☐ With a little difficulty	☐ With some difficulty	☐ With much difficulty	☐ Unable to do
**63. How able is the participant to cut their toenails?**				
☐ Without any difficulty	☐ With a little difficulty	☐ With some difficulty	☐ With much difficulty	☐ Unable to do
**64. How able is the participant to bend down and pick up clothing from the floor?**				
☐ Without any difficulty	☐ With a little difficulty	☐ With some difficulty	☐ With much difficulty	☐ Unable to do
**65. How much DIFFICULTY does the participant currently have using a spoon to eat a meal?**				
☐ No difficulty	☐ A little difficulty	☐ Some difficulty	☐ A lot of difficulty	☐ Can't do

**66. How much DIFFICULTY does the participant currently have putting on a pullover shirt?**				
☐ No difficulty	☐ A little difficulty	☐ Some difficulty	☐ A lot of difficulty	☐ Can't do
**67. How much DIFFICULTY does the participant currently have taking off a pullover shirt?**				
☐ No difficulty	☐ A little difficulty	☐ Some difficulty	☐ A lot of difficulty	☐ Can't do
**68. How much DIFFICULTY does the participant currently have removing wrappings from small objects?**				
☐ No difficulty	☐ A little difficulty	☐ Some difficulty	☐ A lot of difficulty	☐ Can't do
**69. How much DIFFICULTY does the participant currently have opening medications or vitamin containers (e.g., childproof containers, small bottles)?**				
☐ No difficulty	☐ A little difficulty	☐ Some difficulty	☐ A lot of difficulty	☐ Can't do
